# Structural basis for epitope masking and strain specificity of a conserved epitope in an intrinsically disordered malaria vaccine candidate

**DOI:** 10.1038/srep10103

**Published:** 2015-05-12

**Authors:** Rodrigo A. V. Morales, Christopher A. MacRaild, Jeffrey Seow, Bankala Krishnarjuna, Nyssa Drinkwater, Romain Rouet, Robin F. Anders, Daniel Christ, Sheena McGowan, Raymond S. Norton

**Affiliations:** 1 Medicinal Chemistry, Monash Institute of Pharmaceutical Sciences, Monash University, Parkville, VIC 3052, Australia; 2 Department of Biochemistry and Molecular Biology, Monash University, Clayton, VIC 3800, Australia; 3 Garvan Institute of Medical Research, Darlinghurst, Sydney, NSW 2010, Australia; 4 Department of Biochemistry, La Trobe Institute for Molecular Science, Melbourne, VIC 3086, Australia

## Abstract

Merozoite surface protein 2 (MSP2) is an intrinsically disordered, membrane-anchored antigen of the malaria parasite *Plasmodium falciparum*. MSP2 can elicit a protective, albeit strain-specific, antibody response in humans. Antibodies are generated to the conserved N- and C-terminal regions but many of these react poorly with the native antigen on the parasite surface. Here we demonstrate that recognition of a conserved N-terminal epitope by mAb 6D8 is incompatible with the membrane-bound conformation of that region, suggesting a mechanism by which native MSP2 escapes antibody recognition. Furthermore, crystal structures and NMR spectroscopy identify transient, strain-specific interactions between the 6D8 antibody and regions of MSP2 beyond the conserved epitope. These interactions account for the differential affinity of 6D8 for the two allelic families of MSP2, even though 6D8 binds to a fully conserved epitope. These results highlight unappreciated mechanisms that may modulate the specificity and efficacy of immune responses towards disordered antigens.

Intrinsically disordered proteins are highly abundant in *Plasmodium* and related pathogenic genera^1^, and several have been identified as potential vaccine candidates for malaria^2^,^3^,^4^,^5^,^6^,^7^ and other diseases^8^,^9^. For example, the protective effects of RTS,S, the most advanced malaria vaccine in clinical development, appear to be mediated by antibodies to the unstructured repeats of the *Plasmodium falciparum* circumsporozoite protein^6^,^10^. The interactions of intrinsically disordered proteins with their binding partners differ from those of structured proteins in terms of specificity and kinetics^11^, as well as the physicochemical properties of the interacting residues^12^, and are functionally important for a variety of cellular processes^13^. Nonetheless, the role of protein disorder in modulating the immune response against unstructured antigens remains poorly understood.

MSP2 is one of the most abundant and polymorphic glycosylphosphatidylinositol (GPI)-anchored proteins on the surface of the *P. falciparum* merozoite, the invasive blood-stage form of the malaria parasite^14^,^15^. All variants of MSP2 share conserved N- and C-terminal regions but fall into two allelic families, 3D7 and FC27, distinguished by tandem repeats and dimorphic flanking sequences within the central region of the protein^14^,^16^. Human vaccine trial subjects immunized with recombinant 3D7 MSP2 mounted IgG responses capable of recognizing the parasite and significantly reducing parasitemia^17^. However, this vaccine preferentially targeted parasites expressing a 3D7-type MSP2 sequence, indicating that vaccine efficacy was mediated by strain-specific responses to MSP2^18^,^19^. Consistent with this result, the polymorphic region appears to be immunodominant in the natural immune response to MSP2^20^,^21^ and some conserved region epitopes are cryptic on the parasite surface^22^,^23^. Understanding the mechanisms by which these epitopes are masked on the parasite surface should facilitate the design of MSP2-based antigens that direct the human immune response towards conserved epitopes and thus achieve strain-transcending protection.

Here, we use the mouse monoclonal antibody (mAb) 6D8, which recognizes a conserved N-terminal epitope on recombinant MSP2^24^ but does not recognize the parasite surface,^22^ to gain insights into epitope masking and strain specificity of the antibody response to MSP2. Using surface plasmon resonance (SPR) and NMR experiments, we show that recombinant MSP2, when C-terminally anchored to membrane mimetics, adopts a conformation that precludes the binding of mAb 6D8. X-ray crystal structures reveal the structural basis for this epitope masking. In addition, although the 6D8 epitope is fully conserved, its affinity for the antibody is modulated by transient interactions with flanking variable sequences. The ability of a variable region to confer strain specificity on a neighboring conserved epitope has important implications for our understanding of the immunogenic response to disordered vaccine candidates such as MSP2.

## Results

### Lipid interactions block recognition by 6D8

**Figure 1:**
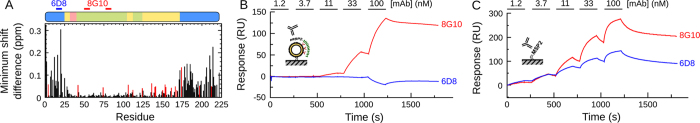
Membrane anchoring recapitulates the solvent exposure of the central variable region and the masking of an N-terminal epitope in recombinant MSP2. (**A)** NMR chemical shift perturbations of His-tagged MSP2 in DOGS-NiNTA/DPC micelles indicate marked differences at the conserved N and C-terminal portions of the protein. Red bars indicate chemical shift perturbations of Asp/Glu residues due to local pH at the micelle surface. Sequence features of MSP2 are shown above, with conserved regions in blue, repeats in green, allele-specific dimorphic regions in yellow and polymorphic regions in pink. (**B)** SPR sensorgrams demonstrating the complete loss of N-terminal recognition of lipid-bound FC27 MSP2 by mAb 6D8 despite the dose-dependent binding of the strain-specific mouse antibody 8G10. The locations of the 6D8 and 8G10 epitopes are indicated in (**A**). (**C**) The binding of mAb 8G10 and mAb 6D8 to MSP2 is concentration dependent in the absence of lipids.

The N-terminal conserved region of MSP2 was shown previously to undergo disorder-to-order transitions in the presence of dodecylyphosphocholine (DPC) micelles^25^,^26^. These interactions, although weak, were sufficient to stabilize the 25-residue N-terminal peptide as an α-helix, spanning at least residues 10–22. The possibility that this helical structure may contribute to epitope masking was explored with full-length MSP2 using a novel proxy of GPI anchoring in which a nickel-chelating lipid was used to bind the C-terminally His-tagged MSP2, mimicking the association of the MSP2 C-terminus with the lipid surface (Fig. 1). A comparison of ^1^H-^15^N HSQC spectra of C-terminally His-tagged FC27 MSP2 in the presence and absence of dodecylphosphocholine (DPC) micelles containing 1 mol % of the nickel-chelating lipid 1,2-di-(9Z-octadecenoyl)-*sn*-glycero-3-[(N-(5-amino-1-carboxypentyl)iminodiacetic acid)succinyl] (DOGS-NTA) revealed substantial changes, involving both line-broadening and chemical shift changes, indicative of extensive interactions between MSP2 and the DPC micelle (Fig. S1). In our previous studies of the N-terminal region of MSP2^25^ we have shown that extensive line-broadening in the presence of high DPC concentrations, as seen here, is preceded at lower DPC concentrations by weaker broadening together with chemical shift changes that are consistent with the adoption of helical structure. In contrast to those studies of the MSP2-DPC interaction in the absence of the DOGS-NiNTA tether, the interactions seen here are not restricted to the N-terminal region of MSP2. In the absence of assignments for the DPC-bound state, we analyzed these changes as minimum chemical shift changes from the DPC-free state (Fig. 1A), calculated from the distance between each assigned peak in the free MSP2 spectrum and the closest peak in the unassigned spectrum of MSP2 in complex with DOGS-NiNTA/DPC as [ΔδH_N_^2^ + (ΔδN/5)^2^]^1/2^. Changes to residues in the variable region of MSP2 are uniformly small and without significant line-broadening, and are restricted to Asp and Glu residues (red bars in Fig. 1A). Because these experiments are performed at pH 4.7, near the expected pK_a_ of these residues, and because the directions of the chemical shift changes are consistent with those we have observed for lipid-free MSP2 upon small changes to the solution pH around this value (data not shown), it is likely that these changes are due to a small change in local pH at the micelle surface. In contrast, perturbations in both the N- and C-terminal conserved regions are larger, with chemical shift changes and extensive line-broadening affecting all residue types. These data indicate that both the N- and C-terminal conserved regions are involved in lipid interactions in this system, consistent with a possible role for these interactions in masking conserved region epitopes.

To test this possibility, we examined antibody binding to lipid-tethered MSP2 by SPR. Vesicles of POPC containing 1 mol% DOGS-NTA were immobilized on an L1 Biacore chip and loaded with Ni^2+^ then C-terminally His-tagged MSP2, and the resulting surface was interrogated for antibody binding. In this assay, mAb 8G10, which recognizes the disordered variable region of FC27 MSP2 on the parasite surface^22^,^27^, bound strongly to vesicles decorated with C-terminally His-tagged FC27 MSP2. In contrast, mAb 6D8, which recognizes an N-terminal conserved epitope on recombinant MSP2^24^ but fails to bind native MSP2 on the parasite surface^22^, did not interact with MSP2 on this artificial lipid surface (Fig. 1B). When MSP2 was immobilized using conventional amide coupling to the SPR chip in the absence of lipid, 6D8 binding was retained (Fig. 1C).

### Determination of the minimal 6D8 epitope

The mAb 6D8 was first demonstrated to bind the conserved N-terminus of MSP2^24^, with the epitope localized to residues 11 to 23 (MSP2_11-23_)^22^. This region of full-length MSP2 undergoes structural changes in the presence of lipids as demonstrated previously^25^,^26^ and confirmed here (Fig. 1A). To explore the structural basis of epitope masking, it was necessary to first define the minimal epitope of mAb 6D8. Six 8-residue peptides were prepared corresponding to a single amino acid frame shift over MSP2_11–23_ (Table. 1) and tested against immobilized mAb 6D8 by SPR. MSP2_15–22_ was the only 8-residue peptide of this initial panel to show detectable binding.

The affinity of MSP2_15–22_ for mAb 6D8 was found to be 87 nM, which is more than ten-fold weaker than for MSP2_11–23_ (6 nM), suggesting that residues beyond the mapped 8-residue region also contribute to antibody recognition. We tested this hypothesis with a second panel of synthetic peptides corresponding to single or double amino acid extensions at the N- and/or C-terminus of MSP2_15–22_ (Table 1). Although a single C-terminal amino acid extension had no effect on binding, a single N-terminal extension was sufficient to restore binding to control levels (6 nM) (Table 1, Fig. S3). Consistent with the results from the 8-residue peptide series, these peptides demonstrate that removal of either Ala15 or Arg22 abolishes binding of MSP2 to 6D8, at up to 1 μM peptide concentration (Table. 1; compare MSP2_15–23_ with MSP2_16–23_, and MSP2_14–21_ with MSP2_14–22_).

### Helical propensity of peptide epitopes

**Figure 2:**
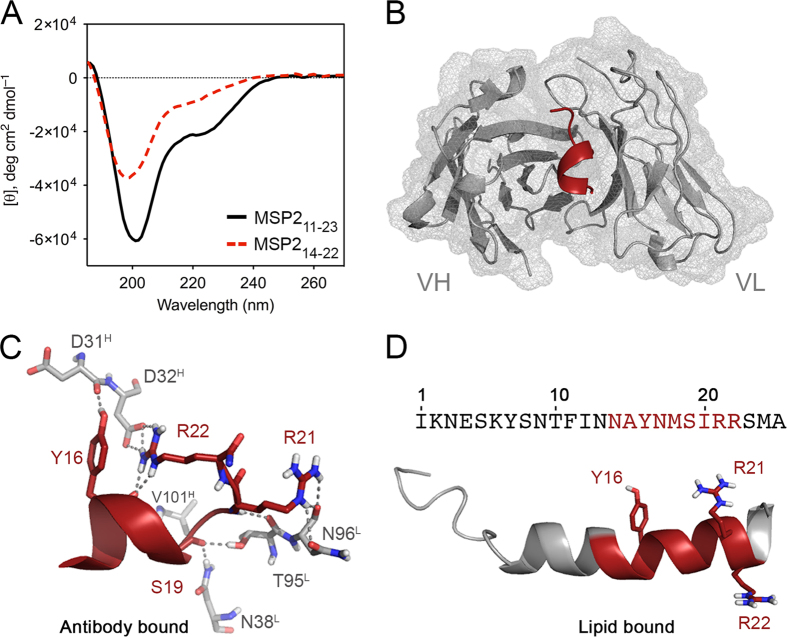
Crystal structure to 1.2 Å resolution of 6D8 Fv bound to the 9-mer peptide MSP2_14–22_. (**A**) Helical propensity of synthetic epitope-bearing peptides MSP2_11–23_ and MSP2_14–22_. Both peptides showed ellipticity at 190, 208 and 220 nm, consistent with some helical content in water, with the longer peptide having significantly greater tendency for helical conformation. (**B**) Crystal structure of MSP2_14–22_ at the paratope cleft formed by the antibody CDRs. (**C**) MSP2_14–22_ adopts a single turn of helix stabilized by hydrogen bonds with residues Asp31^H^, Asp32^H^, Val101^H^ and Asn38^L^. In solution, Arg22 contacts residues Thr91^L^ and Asn92^L^ while the guanidine group of Arg23 of the peptide bends towards the N-terminus of the peptide, establishing critical hydrogen bonds with Asp32 in the VH chain and the carbonyl of Tyr16. (**D**) The structure of the lipid-bound form of MSP2_1–25_^25^ indicating the relative orientation of residues Try16, Arg21 and Arg22. The α-helical configuration of the lipid-bound MSP2_1–25_ removes the backbone flexibility required for Arg22 to access Tyr16, which provides a structural rationale for the lack of binding of mAb 6D8 to MSP2 at the parasite membrane.

We have demonstrated previously that the conserved N-terminal portion of MSP2 is unstructured in solution but adopts an α-helical conformation in the presence of lipids or organic solvents such as TFE^25^,^28^. This region of MSP2 encompasses the entire 6D8 epitope, suggesting that secondary structure formation could play a role in antibody binding^22^. The α-helical propensities of MSP2_11–23_ and the equipotent minimal epitope, MSP2_14–22_, were investigated by circular dichroism . The minimal peptide epitope was substantially less helical than the 13-residue peptide (MSP2_11–23_) in aqueous solution (Fig. 2A) and over a range of TFE concentrations (Fig. S2). However, 6D8 binds these peptides with identical affinity, suggesting that recognition by 6D8 does not depend critically on the extent of helical conformation in the unbound epitope.

### The structure of lipid-bound MSP2 is incompatible with recognition by mAb 6D8

To determine the structural basis for 6D8 epitope masking by lipid interactions, we have solved the X-ray crystal structure of the N-terminal epitope MSP2_14–22_ in complex with the Fv region of mAb 6D8 (6D8 Fv) to 1.2 Å resolution (Fig. 2B). Our results indicate that, despite the moderate α-helical propensity of the 6D8 epitope in MSP2 and the stabilizing effect of lipid interactions^25^, the antibody stabilizes only a single turn of helix in the peptide. This turn of helix spans residues Asn14–Met18, and breaks at Ser19, allowing the carbonyl of this residue to make hydrogen bonds to 6D8 through the side chains of Asn38^L^ and Thr95^L^ (6D8 is numbered according to Kabat^29^, with the chain indicated by a superscript)(Fig 2C). The helix is terminated by an unusually long capping motif involving two side-chain to main-chain hydrogen bonds, one between N^ε^ of Arg22 and the carbonyl of Tyr16 and the other between the hydroxyl of Ser19 and the carbonyl of Ala14. This unusual motif positions Arg22 for simultaneous intra-molecular cation-π interactions with the Tyr16 side chain and an inter-molecular salt bridge with Asp32^H^ (Fig. 2C). The complex is further stabilized by hydrogen bonds between peptide residues Tyr16, Ser19, Arg22 and 6D8 residues Asp31^H^, Val101^H^ and Asn96^L^, respectively, as well as van der Waals interactions involving the hydrophobic MSP2 residues Ala15, Met18 and Ile20.

**Figure 3:**
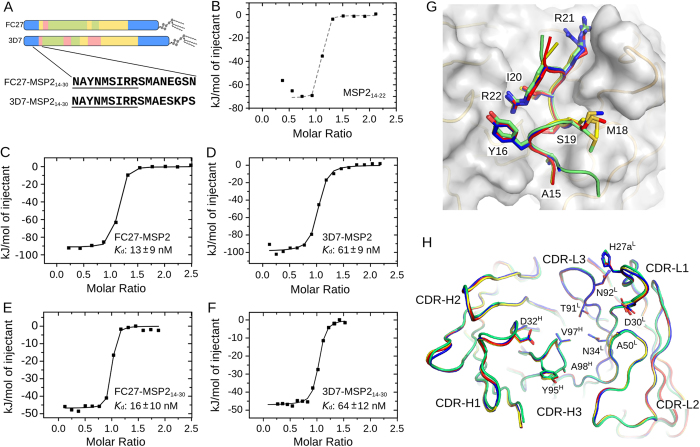
6D8 displays strain-specific binding, even though it recognizes a conserved epitope sequence. (**A**) Schematic of the sequence features of MSP2, showing conserved regions (blue), repeats (green), allele-specific dimorphic regions (yellow) and polymorphic regions (pink), together with the sequences of the two MSP2_14–30_ peptides **b-e**: ITC titrations of MSP2_14–22_ (**B**), full-length FC27 (**C**) and 3D7 (**D**) MSP2, FC27-MSP2_14–30_ (**E**) and 3D7-MSP2_14–30_ (**F**) into 6D8 scFv. Solid black lines in panels C-F are lines of best-fit, with corresponding K_*d*_ values shown with error estimates based on replicate titrations against 6D8 scFv and mAb (n = 3–5). The dashed grey line in panel B illustrates the titration at the limit of determinable affinities (approx. 5 nM under these conditions). (**G**) Superposition of MSP2_14–22_ (blue), MSP2_11–23_ (green), 3D7 MSP2_14–30_ (yellow) and FC27 MSP2_14–30_ (red) in complex with the 6D8 Fv, with a representative molecular surface of the antibody fragment shown. (**H**) Backbone traces of 6D8 Fv bound to MSP2_14–22_ (blue), MSP2_11–23_ (green), 3D7 MSP2_14–30_ (yellow) and FC27 MSP2_14–30_ (red). CDRs and side chains making significant contacts with the antigen are shown.

In contrast to the mostly unstructured MSP2 observed in solution^26^, the N-terminal region of MSP2 is predominantly helical in lipid membranes, with well-defined helical structure extending at least as far as Arg22 (Fig. 2D)^25^,^28^. In the lipid-bound conformation, the side chains of Arg22 and Tyr16 are separated by ~15 Å (Fig. 2D) and are clearly precluded from the interactions jointly made with CDR H1 in the 6D8 complex (Fig. 2C). Similarly, this helical conformation would also prevent the carbonyl of Ser19 from engaging CDRs L1 and L3. Furthermore, lipid interactions are expected to bury the hydrophobic face of this amphipathic helix, preventing access to the key hydrophobic residues that mediate recognition by 6D8, including Tyr16, Met18 and Ile20. The structure of MSP2_14–22_ bound to 6D8 Fv is therefore incompatible with the structure adopted by MSP2 on a lipid surface, which offers a clear explanation for the failure of 6D8 to recognize lipid-bound MSP2. This mechanism of binding was also observed in our 1.7 Å resolution crystal structure of MSP2_11–23_ bound to 6D8 Fv (Fig. 3), with the three-residue N-terminal extension in this peptide making no additional contacts with the antibody. This finding is consistent with the SPR results, which showed that mAb 6D8 binds both peptides with similar affinities (Table 1).

### 6D8 recognizes MSP2 in a strain-specific manner

The interaction between 6D8 and MSP2, was examined further using isothermal titration calorimetry (ITC). The titration of 6D8 scFv with the minimal epitope, MSP2_14–22_, resulted in data at the upper limit of accessible affinities in this system (approximately 5 nM), consistent with the 6 nM K_*d*_ determined by SPR (Fig. 3B). Full-length recombinant MSP2 binds 6D8 scFv with lower affinity, permitting quantification by ITC. Even though 6D8 recognizes a fully conserved epitope in MSP2, the affinities of 6D8 scFv for full-length 3D7 and FC27 MSP2 differ by as much as 5-fold (Fig. 3C,D). To explore the basis of this strain-specificity, we synthesized peptides encompassing the minimal 6D8 epitope and extending C-terminally into the variable region. We found that peptides containing just the first five residues of the variable region of each allele (3D7 and FC27 MSP2_14–30_; Fig. 3A) were sufficient to fully replicate the weaker, strain-specific binding observed for full-length MSP2 (Fig. 3E,F). Identical affinities were observed when 6D8 IgG was used in place of the scFv (Fig. S4).

### Strain-specific recognition is not explained by crystal structures of complexes

In an attempt to determine the structural basis for this strain-specific recognition, we solved the X-ray structure of 6D8 Fv co-crystallized with the strain-specific peptides corresponding to residues 14 to 30 of 3D7 and FC27 (MSP2_14–30_) at resolutions of 1.4 Å and 1.6 Å, respectively. Surprisingly, the basic mode of binding observed in the MSP2_14–22_-6D8 complex was replicated in all complexes crystallized (Fig. 3G,H). The conformation of the MSP2 epitope is identical in all four structures, with backbone RMSD over residues 15–22 of 0.13 Å (heavy atom RMSD 0.54 Å) (Fig. 3G). Similarly, there were no differences in antibody conformation at complementarity determining regions (CDRs) that might offer an explanation for the 10-fold variation in affinity across the peptide-Fv complexes (Figs. 3H & S5).

In the case of FC27-MSP2_14–30_, no interpretable electron density was available to resolve residues C-terminal to Ser24, suggesting that this region fails to make stable interactions with 6D8 Fv, consistent with our determination of MSP2_14–22_ as the minimal 6D8 epitope. The unresolved region includes all of the residues that differ between the FC27 and 3D7 MSP2_14–30_ peptides (Fig. 3A). In contrast, the peptide is resolved until Ser28 in the 3D7-MSP2_14–30_-6D8 Fv complex, although with substantially elevated B-factor values for residues 24–28 (Fig. S6A). In fact, this C-terminal region appears to make only a single significant interaction with the 6D8 Fv, by way of hydrogen bonds between the side chain of Glu27 of 3D7-MSP2_14–30_ and the side chains of Asp52^H^ and Asn55^H^ of 6D8 Fv. Plainly, this interaction depends on one of the carboxylate moieties of Glu27 or Asp52^H^ being protonated. Although this is plausible at the crystallographic pH of 4.2, given the highly solvent-exposed nature of this site it is highly unlikely that the pK_a_ values of these residues are sufficiently perturbed to allow protonation at physiological pH, where our affinity measurements were made. Moreover, this interaction is stabilized by crystal contacts with Arg18^L^ from a crystallographically adjacent 6D8 molecule (Fig. S6B). In NMR spectra recorded at pH 6.5, we see no differences in peaks arising from sidechain NH_2_ groups of 6D8 scFv bound to the peptides 3D7-MSP2_14–30_ and FC7-MSP2_14–30_ (Fig. S6C), suggesting the absence of strain-specific interactions involving Asn55^H^ or other NH_2_ groups under these conditions. Thus, we conclude that the interaction between Glu27 of 3D7–MSP2_14–30_ and Asp52^H^ in 6D8 is an artifact of crystallization. As this is the only crystallographically resolved interaction involving residues that differs between 3D7 and FC27 MSP2, the crystal structures provide no explanation for the observed five-fold difference in affinity.

### Strain-specific recognition is mediated by transient interactions

**Figure 4:**
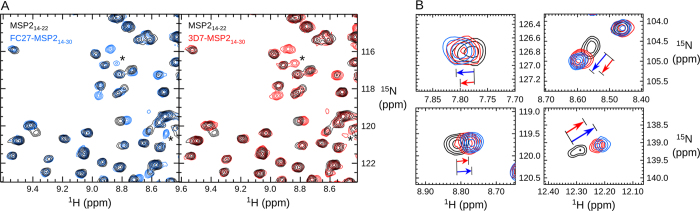
^1^H,^15^N HSQC spectra of 6D8 scFv reveal strain-specific transient interactions. (**A**) Comparison of the spectra of 6D8 in the MSP2_14–22_ complex (black) with the FC27-MSP2_14–30_ complex (blue) and with the 3D7-MSP2_14–30_ complex (red) shows extensive spectral changes. A small number of peaks (*) differ in intensity between the 3D7 and FC27 peptide complex, while others (**B**) undergo chemical shift changes that differ in magnitude, but not direction, between the 3D7 and FC27 peptides as indicated by the arrows.

We therefore turned to NMR spectroscopy to elucidate the basis of the differences in the affinity of 6D8 for 3D7 and FC27 MSP2 (Fig. 4). 6D8 scFv yielded high-quality ^1^H,^15^N HSQC spectra, despite the tendency of scFv to dimerise at high concentrations^30^. Comparisons of the spectrum of 6D8 scFv bound to MSP2_14–22_ with those of 6D8 scFv bound to FC27-MSP2_14–30_ (Fig. 4A & S7) and 3D7-MSP2_14–30_ (Fig. 4B & S7) indicated that the C-terminal extension of the peptide induced significant differences in the spectrum of 6D8, suggesting that MSP2 residues 24 to 30 interact with 6D8 scFv in solution, even though such interactions were not observed crystallographically. Moreover, more subtle differences were observed when the spectra of 6D8 scFv bound to FC27-MSP2_14–30_ and 3D7-MSP2_14–30_ were compared. For some peaks, these differences manifest as differences in intensity (compare peaks marked * in Fig. 4A), while for other peaks small chemical shift differences are observed (Fig. 4B).

Despite the excellent quality of two-dimensional HSQC spectra for these samples, triple-resonance spectroscopy has proven more challenging, presumably due to scFv self-association. For this reason, reliable scFv resonance assignments are not available for any of the 6D8-peptide complexes. While this precludes a direct structural interpretation of the spectral changes observed, analysis of these changes reveals rich variation in the dynamics of these interactions. On the one hand, peaks with variation in intensity without change in chemical shift (such as those marked * in Fig. 4A) are indicative of interactions that are in exchange on a timescale of ms or slower. In this slow exchange case, the variation in peak intensity reflects the population of the exchanging state reported on by that peak.

In contrast, for a subset of peaks (Fig. 4B and Fig. S7), chemical shift changes are observed between the 6D8–MSP2_14–22_ complex and those of the longer peptides. These changes vary in magnitude but not in direction, with changes seen for the FC27 complex being co-linear with, but systematically larger than those seen for the 3D7 complex by approximately 25%. This behavior is most simply explained by fast exchange between two states. In this fast exchange regime the observed chemical shift is a population-weighted average of the chemical shifts of the exchanging states, and thus the chemical shift reports on the relative populations of the exchanging states.. The interactions between 6D8 scFv and MSP2 residues C-terminal to Arg22 therefore include interactions that are exchanging on at least two distinct time-scales, and in which the interacting populations differ between the FC27 and 3D7 peptides. Importantly, all of the chemical shift changes seen here are small, suggesting that these interactions are populated to a relatively small extent, consistent with our inability to resolve these interactions in crystal structures.

## Discussion

Intrinsically disordered proteins, such as MSP2, are abundant components of pathogenic apicomplexan parasites as well as some viruses and other pathogens^1^,^31^. It has been argued that vaccine formulations containing disordered antigens may be advantageous because the effective antibody response against these proteins is less dependent on their native structure^5^. However, disordered proteins often undergo disorder-to-order transitions that are critical for their function and long-range transient interactions that modulate their interactions with binding partners^11^,^13^,^32^. The implications of the conformational dynamics of disordered antigens for the specificity and function of antibodies directed against them are largely unknown, despite posing important challenges for the use of these proteins in vaccine formulations.

During erythrocyte invasion, MSP2 is carried into the invaded cell and rapidly degraded^33^, unlike many merozoite surface antigens, which are released into the blood-stream^34^. As such, interactions between MSP2 and host antibodies occur exclusively at the parasite surface to which MSP2 is attached by a GPI anchor. It has been suggested that local structural changes observed at the N-terminus of MSP2 following lipid interactions^25^ or oligomerization^24^ at the parasite surface may modulate the accessibility of conserved N-terminal epitopes of MSP2. Our results show that a simple membrane-anchoring system employing C-terminally His-tagged MSP2 reproduces the masking of the distant 6D8 epitope on the parasite surface. We demonstrated previously that the N terminal region of MSP2 (MSP2_1–25_) binds lipid, adopting an extended helical conformation spanning at least residues 10–22^25^. The structure of the antibody-bound epitope differs in several important respects from the lipid-bound structure and offers a clear structural explanation for the loss of antibody recognition at the parasite surface. Taken together, our results indicate that lipid interactions alone or in conjunction with the formation of MSP2 oligomers^24^,^35^ play an important role in determining the antigenic characteristics of MSP2 on the merozoite surface^22^.

Coupled folding and binding is often important in the functional interactions of disordered proteins^36^ and has also been demonstrated for disordered antigens^37^,^38^,^39^. Indeed, functional properties of antibodies targeting disordered epitopes have been shown to depend on the specific conformation recognized by these antibodies^38^. The failure of 6D8 to recognize the parasite implies that MSP2 on the merozoite surface, in contrast to recombinant MSP2 in solution, lacks the conformational flexibility required to adopt the conformation bound by the 6D8 antibody. Human monoclonal antibodies targeting the conserved regions of MSP2 have not been characterized to date and polyclonal responses to those regions are significantly underrepresented in patients when compared to variable region antibodies^40^,^41^. Nonetheless, antibodies able to recognize the N-terminal region of MSP2 on the merozoite surface have been characterized^23^,^33^. These antibodies appear to recognize epitopes that overlap the 6D8 epitope characterized here, and as such it is likely that these antibodies recognise this region in a distinct conformation, more compatible with the structure of MSP2 on the parasite surface. Engineering the N-terminal region to favor this mode of antibody recognition could provide a route for the development of a strain-transcending MSP2 vaccine.

While disordered proteins often adopt an ordered conformation when interacting with binding partners, significant disorder can persist in these complexes^13^,^42^. Transient or ‘fuzzy’ interactions allow disordered epitopes to interact with multiple partners in biological roles otherwise not accessible to structurally rigid proteins^13^,^32^,^43^,^44^. However, these interactions have not, to our knowledge, been implicated in the modulation of antibody recognition. Here we have shown that transient interactions involving residues outside the structurally defined epitope of mAb 6D8 confer strain-specificity on the recognition of MSP2 by this antibody. ITC data demonstrate that a C-terminal extension from the minimal 6D8 epitope reduces the affinity of the complex, presumably as a consequence of the entropic costs associated with the restriction of conformational freedom that accompanies antibody binding. This entropic cost is partially offset by transient, fuzzy interactions^13^, shown by NMR to occur over a range of time-scales, between the Fv domain and regions of MSP2 immediately C-terminal to the defined epitope. These interactions differ in extent between 3D7 and FC27 MSP2, thus accounting for the difference in affinity of mAb 6D8 for the two allelic forms.

The retention of proteins of low sequence complexity in apicomplexan genomes has long been postulated as an adaptive strategy against the host’s immune system^45^,^46^,^47^. This hypothesis has been challenged because the majority of disordered apicomplexan proteins are not antigenic^48^. However, recent work has recognized distinct types of low-complexity sequence within the *P. falciparum* genome, one class of which includes several important antigens including MSP2^49^. Our finding that antibody recognition of a conserved epitope can be strain-specific as a consequence of transient interactions between antibody and antigen highlights a potentially important but largely unappreciated mechanism of immune evasion in this class of proteins. In disordered antigens, such as MSP2, conserved epitopes are flanked by disordered polymorphic regions^1^. Transient interactions between these regions and host antibodies may explain the difficulty in establishing broad neutralizing responses to conserved antigens and shed light on the possible benefit of these regions for the survival of *P. falciparum* in the human host.

## Materials and methods

### Recombinant protein expression

The two allelic forms of MSP2 were prepared as described previously^26^,^50^. Soluble antibody fragments assembled as heterodimeric (Fv) and monomeric (scFv) polypeptide chains using 6D8 VH and VL sequences (Fig. S8) were produced in *Escherichia coli* and affinity purified using 3D7-MSP2- coated beads (Fig. S9) as described in the SI Materials and methods.

### Affinity measurements

The affinity of 6D8 IgG and scFv for synthetic peptides and recombinant MSP2 was determined by ITC (Microcal ITC-200, GE Healthcare) and SPR (Biacore T200, GE Healthcare) using a Mouse Antibody Capture kit (GE Healthcare) (SI Materials and methods). Antibody binding to lipid-tethered MSP2 was assessed SPR using an L1 chip (GE Healthcare). Vesicles of POPC containing 1 mol % DOGS-NTA were adsorbed onto the chip, and loaded with Ni^2+^ and then FC27 MSP2–6His.

### X-ray data collection and structure refinement

Complexes of 6D8 Fv with synthetic peptides corresponding to the conserved N-terminal regions MSP2_11–23_ and MSP2_14–22_ and the allele-specific N-terminal peptides 3D7 MSP2_14–30_ and FC27 MSP2_14–30_ were crystallized by the hanging-drop method under the conditions listed in Table S1 (SI Materials and methods). Structures were solved by molecular replacement to resolutions ranging from 1.2 Å to 1.7 Å for the different complexes. Refinement statistics are provided in Table S2. The presence and identity of the peptide in each 6D8 crystal was confirmed by LC-MS.

### NMR spectroscopy

^13^C, ^15^N-labelled 6D8 scFv was dissolved in 20 mM sodium citrate, pH 6.5, 7% ^2^H_2_O, in the absence of ligand to a concentration of approximately 100 μM. Peptides were added to 20% molar excess. ^15^N-labelled FC27-MSP2-6His was dissolved to 200 μM concentration in 20 mM NaAcOH, pH 4.7 containing 100 mM DPC, 1 mM DOGS-NTA and 1mM NiCl_2_. ^1^H-^15^N HSQC spectra were recorded at 35 °C on a 600 MHz Bruker Avance III spectrometer equipped with a TCI triple-resonance cryoprobe. Spectra were processed using Topspin 3.2 (Bruker).

## Additional Information

**How to cite this article**: Morales, R. A. V. *et al*. Structural basis for epitope masking and strain specificity of a conserved epitope in an intrinsically disordered malaria vaccine candidate. *Sci. Rep.* **5**, 10103; doi: 10.1038/srep10103 (2015).

## Supplementary Material

Supplementary Information

## Figures and Tables

**Table 1:** Mapping of the optimal binding region of the mAb 6D8 on the MSP2_11-23_ sequence using a panel of overlapping synthetic peptides.

Peptide	Sequence∆	*K*_d_ (nM)^ø^
MSP2_11-23_*	FINNAYNMSIRRS	6±3
MSP2_11-18_	FINNAYNM	>1000
MSP2_12-19_	INNAYNMS	>1000
MSP2_13-20_	NNAYNMSI	>1000
MSP2_14-21_	NAYNMSIR	>1000
MSP2_15-22_	AYNMSIRR	87±21
MSP2_16-23_	YNMSIRRS	>1000
MSP2_15-23_	AYNMSIRRS	34±12
MSP2_14-22_	NAYNMSIRR	6±2
MSP2_13-22_	NNAYNMSIRR	14±5
MSP2_14-23_	NAYNMSIRRS	16±7

øK_*d*_ determined by SPR ± standard deviation of three replicate experiments.

^∆^Synthetic peptides were N-terminally acetylated and C-terminally amidated;

^*^Broad epitope mapped previously^22^
