# Supplementary material for: Structural basis for epitope masking and strain specificity of a conserved epitope in an intrinsically disordered malaria vaccine candidate
Source: Sci Rep. 2015 May 12;5:10103. doi: 10.1038/srep10103 (PMC4428071; doi:10.1038/srep10103)
Supplement: Supplementary Information [file srep10103-s1.pdf]

Supplementary Information

Structural basis for epitope masking and strain specificity of a conserved epitope in an intrinsically disordered malaria vaccine candidate

Rodrigo A. V. Morales[1], Christopher A. MacRaild[1], Jeffrey Seow[1], Bankala Krishnarjuna[1], Nyssa Drinkwater[2], Romain Rouet[3], Robin F. Anders[4], Daniel Christ[3], Sheena McGowan[2] and Raymond S. Norton*[1]

1. Medicinal Chemistry, Monash Institute of Pharmaceutical Sciences, Monash University, Parkville, VIC 3052, Australia

2. Department of Biochemistry and Molecular Biology, Monash University, Clayton, VIC 3800, Australia

3. Garvan Institute of Medical Research, Darlinghurst, Sydney, NSW 2010, Australia

4. Department of Biochemistry, Institute for Molecular Science, La Trobe University Melbourne, VIC 3086, Australia

* Corresponding author (Ray.Norton@monash.edu)

Supplementary Methods

Recombinant expression and purification of 3D7 and FC27 MSP2. Two allelic forms of 3D7 and FC27 MSP2 (Genebank accession numbers JN248383 and JN248384, (http://www.ncbi.nlm.nih.gov)) were produced recombinantly in *Escherichia coli* BL21(DE3) Gold (Stratagene) cells under different strategies. FC27 MSP2 was expressed in high yield without tags or carrier proteins, as described,[1,2] but this strategy was not useful for 3D7 MSP2 owing to poor expression yields. Optimal expression of 3D7 MSP2 was achieved using a codon-optimised construct (Life Technologies) in a thioredoxin-6xHis expression system (pET32a, Millipore Pty) as described in (MacRaild *et al.* in press).

Recombinant expression and purification of anti-MSP2 antibody fragments. Variable heavy and light chain sequences corresponding to the anti-MSP2 6D8 mAb were PCR-amplified directly from the 6D8 mouse hybridoma cell line[3,4] using sequence-specific primers.[5] Primer-derived errors found at the first conserved N-terminal residues of the VH sequence were corrected based on the closely-related V_H mouse germline, IGHV1-78 (http://www.imgt.org/) (Fig. S8). Variable heavy (V_H) and light (V_L) chains are available under accession codes KM393285 and KM393286, respectively.

Soluble antibody fragments were assembled as heterodimeric (Fv) and monomeric (scFv) polypeptide chains using 6D8 VH and VL sequences optimised for expression in *E. coli* BL21-Gold as described previously.[6] Synthetic genes were purchased from Genscript and cloned into pET12a (Merck Millipore) for periplasmic expression in *E. coli* were grown in presence of tetracycline (15 µg/mL) and ampicillin (100 µg/mL). Protein expression was carried out under IPTG induction for 20 h at room temperature in LB or M9 minimal media supplemented with ^{15}N-ammonium chloride and ^{13}C-glucose at 1 g/L and 4 g/L respectively for isotopic labeling.[7] Periplasm contents were extracted with gentle treatment of the cell mass with sucrose buffer (100 mM Tris-HCl, 1 mM EDTA and 20% sucrose (w/v) pH 8.0) followed by 5 mM $MgCl_2$ at 4 ºC.

The 6D8 antibody fragments were affinity purified from the periplasmic fraction using 3D7 MSP2-bound beads prepared by incubation of full-length recombinant 3D7 MSP2 with hydroxylsuccimidyl-coated Sepharose beads (Sigma-Aldrich) according to the manufacturer's instructions. Bound antibody fragments were washed extensively with 50 mM phosphate buffer pH 7 and eluted from the MSP2 beads with 100 mM glycine, pH 2.7. Samples were neutralized with 1M Tris, concentrated in a 10 kDa MWCO centrifuge filter (AMICON Ultra-0.5, Merck-Millipore) and dialyzed against 50 mM ammonium bicarbonate prior to lyophilization and storage at -80 ºC. The purity of each antibody fragment was assessed by SDS-PAGE and LC-MS (Fig. S9).

Peptide synthesis. Standard FMOC-protected amino acids (Phe, Ile, Asn(Trt), Ala, Tyr(tBu), Met, Ser(tBu) and Arg(Pbf)), Rink amide polystyrene resin and O-(1H-6-chlorobenzotriazol-1-yl)-N,N,N',N'-tetramethyluronium hexafluorophosphate (HCTU) were obtained from Chem-Impex. Organic solvents dimethylformamide (DMF), acetonitrile and diethyl ether were obtained from Merck Pty. Trifluoroacetic acid (TFA) was obtained from Peptides International. Piperidine, diisopropylethylamine (DIPEA), triisopropylsilane (TIPS) and acetic anhydride were obtained from Sigma-Aldrich.

A panel of synthetic peptides corresponding to the conserved N-terminal regions $MSP2_{11-23}$, $MSP2_{11-18}$, $MSP2_{12-19}$, $MSP2_{13-20}$, $MSP2_{14-21}$, $MSP2_{15-22}$, $MSP2_{16-23}$, $MSP2_{14-23}$, $MSP2_{14-22}$, $MSP2_{13-23}$, $MSP2_{14-23}$ and the allele-specific regions 3D7 $MSP2_{14-30}$ and FC27 $MSP2_{14-30}$ were prepared in-house using standard 9-fluorenylmethoxycarbonyl (FMOC) solid-phase chemistry.[8] Briefly, MSP2 peptides were assembled over Rink amide polystyrene resin (0.1

mM scale) using a three-fold equivalent of FMOC-protected amino acid, HCTU and DIPEA in DMF per cycle. Each amino acid coupling was carried out for 30 min at room temperature with continuous shaking. Chain deprotection was carried out in 50% piperidine in DMF for 2 min. Each step in the cycle was terminated with an extensive DMF wash. Peptides were N-terminally acetylated after the last coupling and deprotected, using a three-fold equivalent of acetic anhydride and Dipea in DMF.

The fully assembled peptides were dried under vacuum and cleaved for 3 h in a mixture of TFA:TIPS:water (95:2.5:2.5 v/v). The cleaved material was precipitated in cold diethyl ether overnight at -20 ºC. The insoluble peptide material was spun down at 4,000 rpm for 30 min at 4 ºC and the pellet washed twice in cold diethyl ether prior to removal of the organic phase. The crude peptide mixture was resuspended in 50% acetonitrile/0.1% TFA, filtered and freeze-dried prior to HPLC purification. All MSP2 peptides with the exception of $MSP2_{12-19}$ and $MSP2_{13-20}$ were resuspended in solvent A (0.1% TFA in water) to a final concentration of 2 mg/mL for HPLC purification. Peptides $MSP2_{12-19}$ and $MSP2_{13-20}$ showed a high tendency to aggregate and had to be resuspended in 10 mM acetic acid at concentrations of 1 mg/mL. MSP2 peptides were purified on a reverse-phase C18 column (Zorbax, 10 x 300 mm) using a linear gradient of 5 to 40% of solvent B (90% acetonitrile / 9.9% water / 0.1% TFA) against solvent A (0.1% TFA in water) over 40 min. The purity of MSP2 peptides was assessed by mass spectrometry (LC-MS).

Affinity measurements. Binding affinity constants of 6D8 antibodies (IgG and scFv) for synthetic peptides and recombinant allelic forms of MSP2 were determined by isothermal titration calorimetry (Microcal ITC-200, GE Healthcare) using either murine mAb 6D8 produced and affinity-purified by the Walter and Eliza Hall Institute antibody facility[3] or 6D8 scFv. Titrations were performed in 20 mM sodium phosphate, 150 mM NaCl, pH 7.4, at 25°C. Typical antibody concentrations were 10 μM (IgG) or 20 μM (scFv), with MSP2 or peptide titrated from 200 μM stocks. Control titrations of MSP2 into buffer were performed, and the resulting heats of dilution subtracted from the corresponding titration into antibody. Where low peptide solubility precluded ITC measurements, affinity was estimated by surface plasmon resonance (SPR) (Biacore T200, GE Healthcare) using a Mouse Antibody Capture kit (GE Healthcare). Synthetic peptides were resuspended in running buffer (20 mM HEPES, 150 mM NaCl, 3 mM EDTA, 0.05 % Tween20, pH 7.4) and injected over the 6D8-captured surface. Binding affinity was estimated from the concentration dependence of steady-state responses observed.

Antibody binding to lipid-bound MSP2. For NMR samples, micelles of dodecylphosphocholine (DPC) were prepared at 100 mM in 20 mM NaAcOH, pH 4.7 and doped with 1 mol % 1,2-di-(9Z-octadecenoyl)-*sn*-glycero-3-[(N-(5-amino-1-carboxypentyl)iminodiacetic acid)succinyl] (DOGS-NTA) (Avanti Polar Lipids). $NiCl_2$ was added to 1 mM and 7 % ^2H_2O added, and this solution was used to dissolve ^{15}N-FC27-MSP2-6His to a final concentration of 0.2 mM. ^1H-^{15}N SOFAST HMQC spectra[9] were recorded at 25°C on a 600 MHz Bruker Avance III spectrometer equipped with a TCI triple-resonance cryoprobe. Spectra were processed using Topspin (Bruker). Minimum chemical shift differences are calculated from the distance between each assigned peak in the free MSP2 spectrum and the closest peak in the unassigned spectrum of MSP2 in complex with DOGS-NiNTA/DPC as $[\Delta\delta H_N^2 + (\Delta\delta N/5)^2]^{1/2}$.

For SPR assays of antibody binding, lipid vesicles were prepared from 1-palmitoyl-2-oleoyl-*sn*-glycero-3-phosphocholine (POPC) and DOGS-NTA at a 100:1 molar ratio. The lipids were mixed in chloroform:methanol (1:1), dried, and resuspended to approximately 5 mM lipid in buffer (20 mM HEPES, 150 mM NaCl, pH 7.4), then sonicated until a stable,

optically clear solution was obtained. Freshly-prepared vesicles were coated onto reference and active cells of a Biacore L1 chip (GE Healthcare) by injection of a 0.5 mM stock at 2 μL/min for 15 min, washed with a 30 s pulse of 50 mM NaOH (30 μl/min) and loaded with Ni^{2+} with a 3 min injection of 50 mM $NiSO_4$ (2 μL/min). The prepared surface was rinsed for 2 min (30 μL/min running buffer) before MSP2 was loaded onto the lipid surface of the active cell with a 5 min injection of 1.5 μM FC27 MSP2-6His at 2 μL/min. Finally, the surface was allowed to stabilize under flow (30 μL/min running buffer) for 10 min before antibody binding was assessed, typically with five 3-min injections of increasing antibody concentration. The chip surface was regenerated with a 30 s injection of 30 mM NaOH in 40 % (v/v) isopropanol between cycles. In control experiments, identical injections of mAb were performed over MSP2 immobilized to a comparable level on a CM5 chip by amide coupling following the manufacturer's instructions.

Antibody-peptide co-crystallization, X-ray data collection and structure refinement. Crystals of antibody-peptide complexes were obtained using 6D8 Fv bound to synthetic MSP2 peptides corresponding to the conserved N-terminal region $MSP2_{14-22}$ and $MSP2_{11-23}$ and the allele-specific N-terminal regions 3D7 $MSP2_{14-30}$ and FC27 $MSP2_{14-30}$. Antibodies and synthetic peptides were conjugated at 1:1.5 (mol/mol) ratio in crystallization buffer (20 mM Tris-HCl, 100 mM NaCl, pH 7.0) for 1 h at room temperature. The unbound peptide and high molecular weight aggregates were removed by gel filtration (Superdex 75 10/300 GL, GE Healthcare). The complex was concentrated to 20-30 mg/mL by gentle centrifugation (Amicon Ultra 3 kDa, Merck-Millipore Pty) and cleared of precipitants by centrifugation at 10,000 rpm for 10 min, then filtered through a 0.22 μm spin filter at 4ºC. Antibody concentrations were determined based on the absorbance at 280 nm ($A^{1\%}_{280nm}$ = 1.6) by NanoDrop (Thermo-Fisher Pty Ltd).

The crystal complexes were grown using the hanging drop vapour diffusion method, with 1:1 (v/v) ratio of protein to mother liquor (0.5 mL well volume). Large, plate-shaped crystals appeared overnight in the presence of polyethylene glycol 8000, sodium acetate buffer and sodium chloride as shown in Supplementary Table S2. Crystals were cryo-protected by the addition of 10 % glycerol prior to data collection. Datasets were collected at 100 K at the Australian Synchrotron Macro crystallography MX1 beamline 3BM1 and Micro crystallography MX2 beamline 3ID1 at resolutions ranging from 1.2 Å to 1.7 Å for the different complexes. Diffraction images were processed using XDS [10] and Aimless from the CCP4 suite.[11] 5% of each dataset was flagged for calculation of R_{free}[12] with neither a sigma nor a low-resolution cut-off applied to the data. A summary of statistics is provided in Supplementary Table S3.

Structure determination proceeded using the Molecular Replacement (MR) method and the program PHASER.[13] An initial search model was constructed from the crystal structure of a mouse single chain Fv (PDB ID 3GM0) by removing the ligand from the search model. The 6D8 Fv + $MSP2_{14-22}$ dataset showed that a single clear peak was evident in both the rotation and translation functions and packed well within the asymmetric unit. Together with the unbiased features in the initial electron density maps, the correctness of the MR solution was confirmed. Initial electron density maps also clearly showed unbiased features of the $MSP2_{14-22}$ peptide ligand between the two protein chains. Automated model building was performed using the program ARP/warp.[14] All subsequent model building and structural validation was undertaken using Phenix[15,16] and COOT.[17] Solvent molecules were added only if they had acceptable hydrogen-bonding geometry contacts of 2.5 to 3.5 Å with protein atoms or with existing solvent and were in good $2F_o$-F_c and F_o-F_c electron density.

The remaining three structures (6D8 Fv + $MSP2_{11-23}$, 6D8 Fv + 3D7 $MSP2_{14-30}$ and 6D8 Fv + FC27 $MSP2_{14-30}$) were solved using the model of 6D8 Fv alone (excluding ligand and

solvent) as the MR probe. Hydrogen bonds (excluding water-mediated bonds) and salt bridges were calculated using PDBePISA.[18]

Circular dichroism. The secondary structure of the MSP2 peptides $MSP2_{11-23}$ and $MSP2_{14-22}$ was determined by CD spectroscopy. CD spectra were acquired between 185 and 280 nm at 25 ºC on a Jasco J-815 spectropolarimeter (Jasco, ATA Scientific, Japan) using a 1-mm pathlength quartz cell (Starna, Hainault, United Kingdom). Peptide stock solutions were made to a concentration of 100 μM in 10 mM acetic acid. Peptides were tested at a final concentration of 50 μM with increasing amounts of trifluoroethanol (Sigma) (0, 10, 20 and 30% v/v). Spectra were acquired at a rate of 50 nm/min with 1 nm data intervals, 4 s integration time and a 1-nm slit width. Three accumulations were made and averaged to reduce the noise. Signal was recorded in millidegrees and later converted to ellipticity values [θ] using the formula $[\theta] = (\theta \text{ x } R_w / L \text{ x } C)$, where θ is the recorded ellipticity in millidegrees, R_w is the mean residue weight, L is the pathlength in millimeters and C is the concentration in mg/mL.

Table S1. Conditions used for the crystallization of antibody-peptide complexes.

Crystal	Crystallisation Conditions	Dataset Resolution (Å)
6D8 Fv + MSP2$_{14-22}$	22 % (w/v) PEG8000<br>0.1 M sodium acetate (pH 4.7)<br>0.2 M sodium chloride	1.2
6D8 Fv + 3D7 MSP2$_{14-30}$	30 % (w/v) PEG8000<br>0.1 M sodium acetate (pH 4.2)<br>0.2 M sodium chloride	1.4
6D8 Fv + FC27 MSP2$_{14-30}$	30 % (w/v) PEG8000<br>0.1 M sodium acetate (pH 4.4)<br>0.2 M sodium chloride	1.6
6D8 Fv + MSP2$_{11-23}$	20 % (w/v) PEG8000<br>0.1 M sodium acetate (pH 4.2)<br>0.2 M sodium chloride	1.7

Supplementary Table S2. Data collection and refinement statistics.

Data collection	6D8 Fv + $MSP2_{14-22}$	6D8 Fv + 3D7 $MSP2_{14-30}$	6D8 Fv + FC27 $MSP2_{14-30}$	6D8 Fv + $MSP2_{11-23}$
Space Group	P21	P2	P2	P212121
Cell dimensions (Å)	a=41.9<br>b=60.7<br>c=43.7,<br>α = 90°; β = 106.7°; γ = 90°	a=42.1<br>b=60.3<br>c=43.5<br>α = 90°; β = 107.0°; γ = 90°	a=42.1<br>b=60.2<br>c=43.4<br>α = 90°; β = 107.1°; γ = 90°	a=35.2<br>b=64.3<br>c=89.9<br>α = β = γ = 90°
Resolution (Å)	41.83 – 1.21 (1.27 – 1.21)	34.39 – 1.35 (1.43 – 1.35)	34.40 – 1.58 (1.67 – 1.58)	52.3 – 1.7 (1.76 – 1.7)
Total reflections	1560904	258993	213302	315156
Unique reflections	62158	44204	28505	23236
Multiplicity	25.1 (3.6)	5.9 (3.5)	7.5 (3.6)	13.6 (12.3)
Data Completeness (%)	96.8 (83.3)	97.4 (95.8)	99.9 (99.7)	100.0 (100.0)
<I/s_I>	32.5 (1.7)	16.3 (2.1)	21.1 (6.8)	9.4 (3.0)
CC(1/2)	0.904 (0.578)	0.999 (0.707)	0.998 (0.971)	0.991 (0.680)
Rpim (%)[b]	6.4 (51.9)	22 (366)	21 (96)	17.0 (148.0)
PDB	4QYO	4QY8	4QXT	4R3S
Structure refinement				
Non hydrogen atoms				
Protein	1821	1829	1808	1847
Solvent (HOH)	377	369	326	370
R_{free} (%)	17.0	16.5	17.8	20.0
R_{cryst} (%)	15.1	15.2	15.6	15.2
CC*	0.974	0.906	0.916	0.735
Bond lengths (Å)	0.008	0.005	0.008	0.017
Bond angles (°)	1.25	1.10	1.18	1.61
Ramachandran plot				
Favoured (%)	98	97	98	98
Outliers (%)	-	-	-	-
B factors ($Å^2$)				
Mean protein	13.5	15.7	17.0	9.10
Mean water molecule	30.6	29.3	32.0	23.8
Molprobity Score[c]	1.12<br>97th percentile (N= 1422, 1.208 Å ± 0.25 Å)	0.93<br>100th percentile (N =2978, 1.353Å ± 0.25Å)	0.76<br>100th percentile (N =6761, 1.580Å ± 0.25Å)	1.24<br>97th percentile (N= 9248, 1.700 Å ± 0.25 Å)

a Values in parentheses refer to the highest resolution shell.
b Agreement between intensities of repeated measurements of the same reflections and can be defined as: ∑(Ih,i – <Ih>)/∑ Ih,i, where Ih,i are individual values and < Ih > is the mean value of the intensity of reflection h.

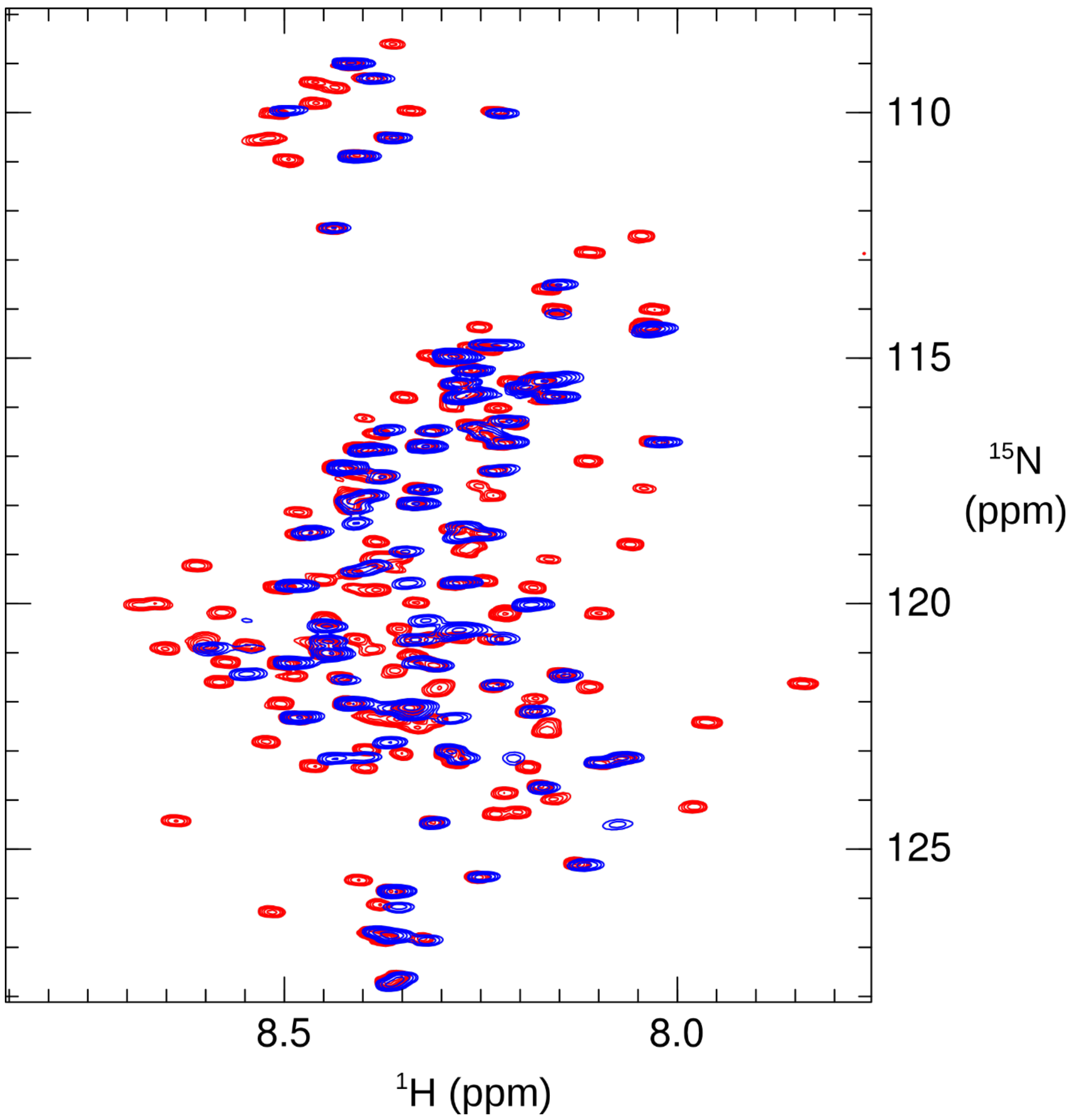

Figure S1. ^{1}H-^{15}N SOFAST-HMQC spectra of FC27-MSP2-6His in the presence (blue) and absence (red) of dodecylphosphocholine (DPC) micelles containing 1 mol % of the Ni^{2+}-charged chelating lipid 1,2-di-(9Z-octadecenoyl)-*sn*-glycero-3-[(N-(5-amino-1-carboxypentyl)iminodiacetic acid)succinyl (DOGS-NTA).

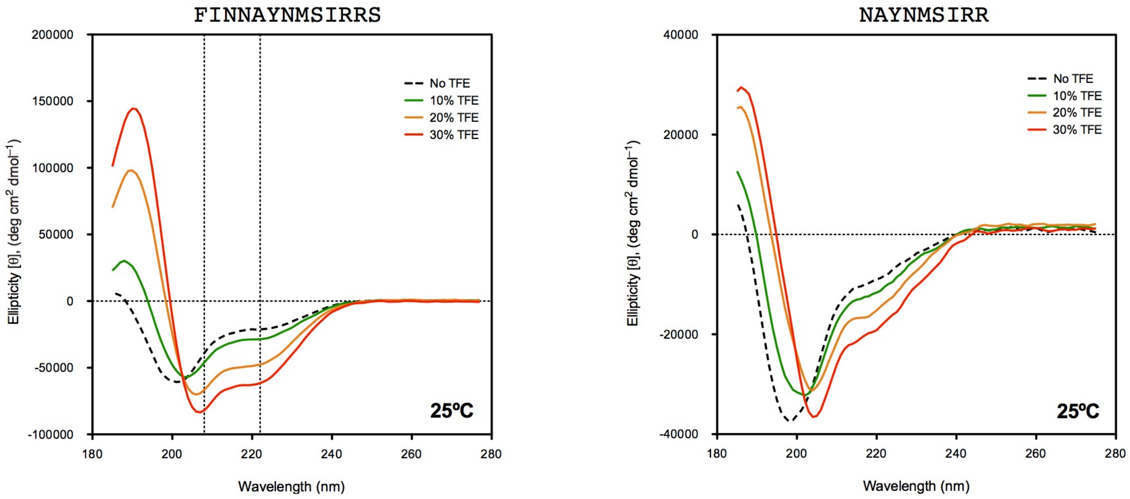

Figure S2. Helical propensity of synthetic epitope-bearing peptides MSP2$_{11-23}$ and MSP2$_{14-22}$. Both peptides showed decreases in ellipticity at 208 and 220 nm and an increase in peak height at 190 nm, characteristic of α-helical motifs adopted upon trifluoroethanol addition (0 to 30%). The minimal peptide epitope MSP2$_{14-22}$ was less helical than the 13-mer peptide (MSP2$_{11-23}$) as expected for such short peptides. However, a helical component present in both peptides is small but still prone to stabilisation in lipid-like environments.

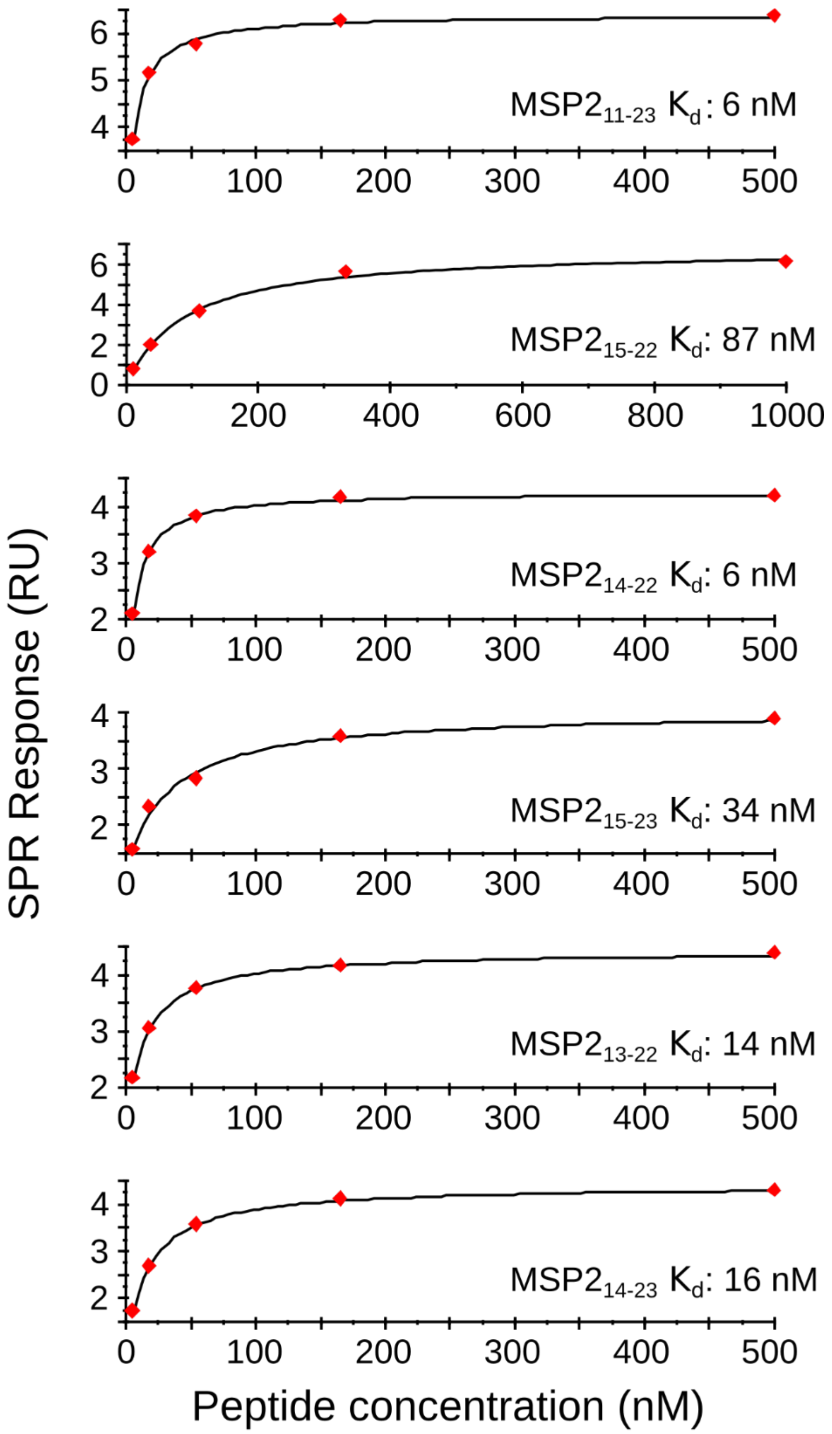

Figure S3. Representative SPR data for the determination of peptide binding affinities for immobilised 6D8.

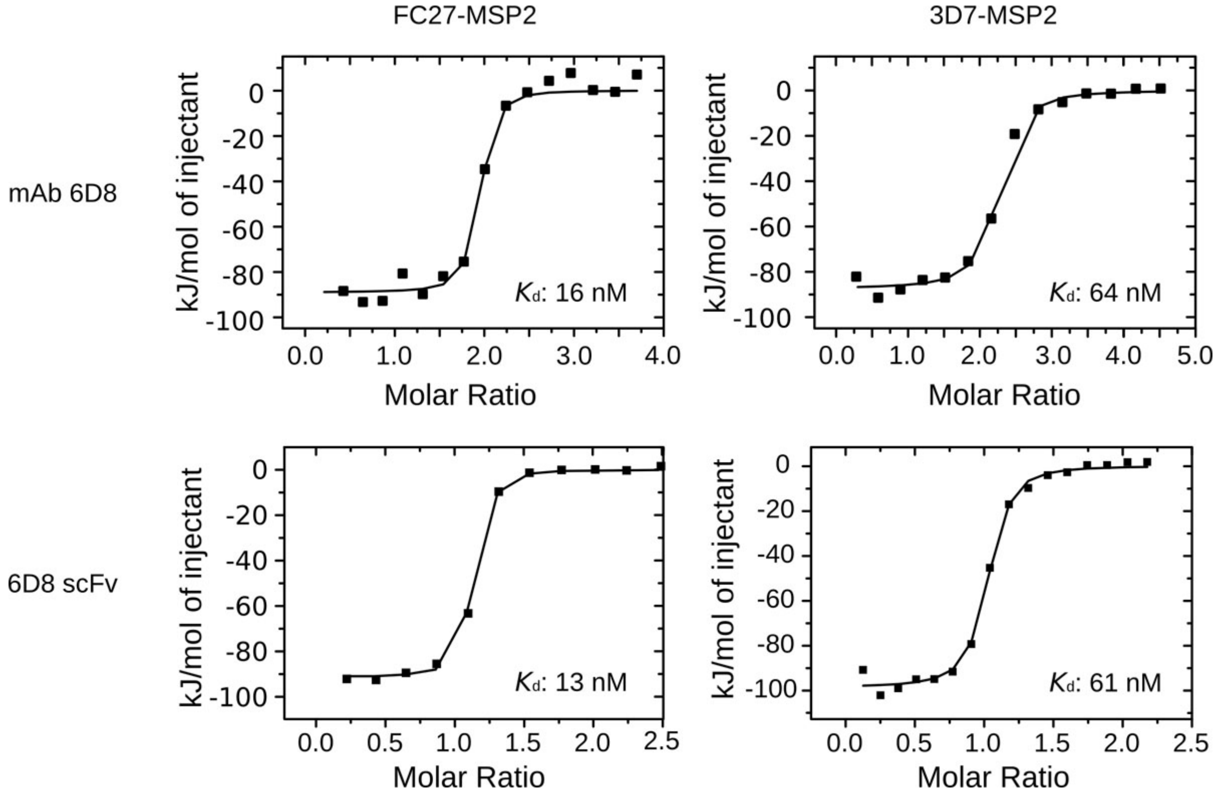

Figure S4. ITC titration of MSP2 to the mAb 6D8 (IgG) and 6D8 scFv. Binding data indicate that recognition of full-length 3D7 and FC27 MSP2 alleles is unaffected by the conversion of the full-length IgG antibody into a single-chain fragment. The strain-selectivity observed in 6D8 IgG is also fully reproduced in the scFv.

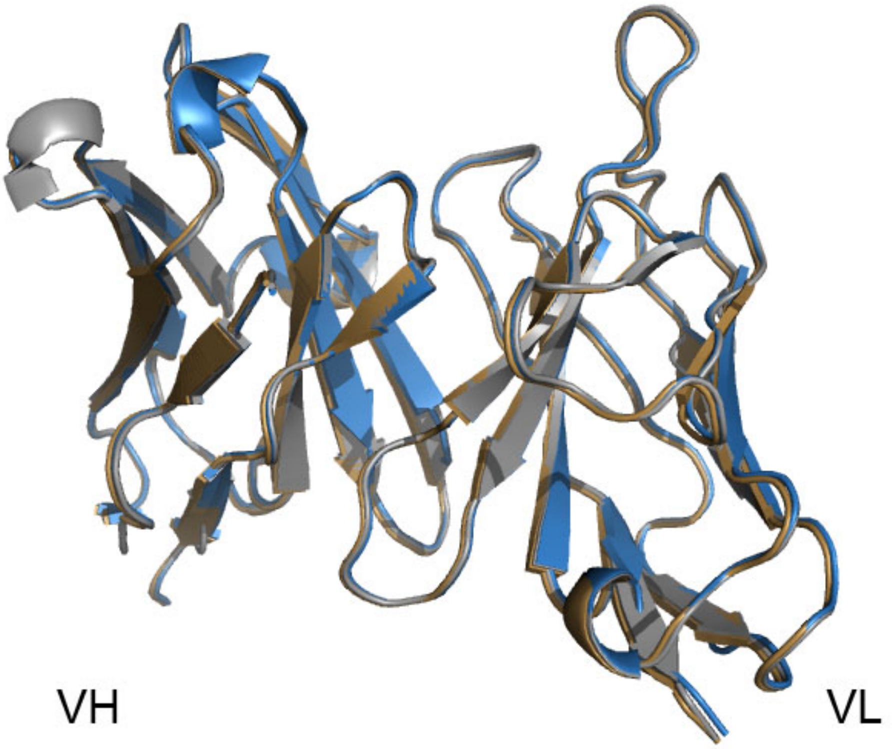

Figure S5. Overlay of the bound antibody structure of crystal complexes 6D8 Fv-MSP2$_{14-22}$, 6D8 Fv-3D7 MSP2$_{14-30}$ and 6D8 Fv-FC27 MSP2$_{14-30}$ shown in grey, blue and brown respectively. Despite marked differences in affinity, side-chain deviations across the three crystal structures were minimal and unlikely to explain affinity differences (Cα RMSD of 1.55 Å between 6D8 Fv-MSP2$_{14-22}$ and 6D8 Fv-3D7 MSP2$_{14-30}$ complexes and Cα RMSD of 1.53 Å between 6D8 Fv-MSP2$_{14-22}$ and 6D8 Fv-FC27 MSP2$_{14-30}$).

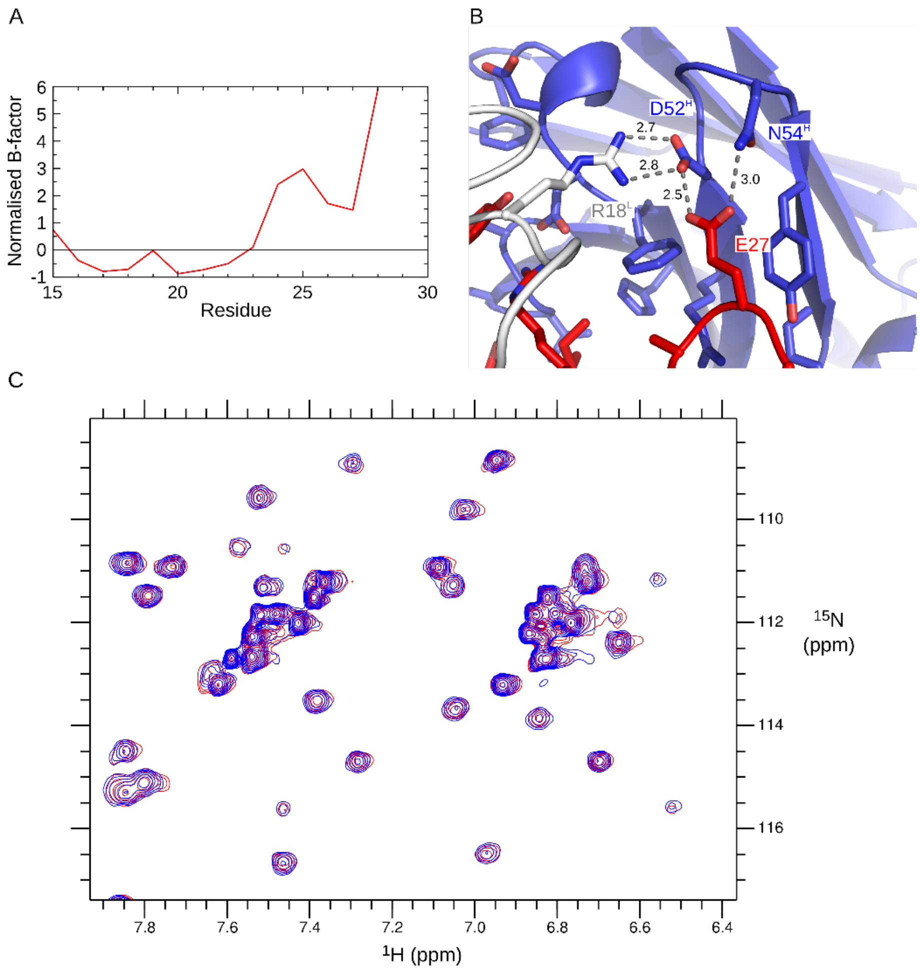

Figure S6. Crystal-contact-mediated interactions of 3D7-MSP2$_{14\text{-}30}$ with 6D8. (**A**). Normalised B-factors of Cα atoms of 3D7-MSP2$_{14\text{-}30}$ in complex with 6D8 Fv. Isotropic B-factors are normalized as (B - <B>)/σ where <B> and σ are the average and standard deviation, respectively, of all Cα B-factors in the complex. (**B**). H-bonds between 3D7-MSP2 residue E27 (red) and 6D8 V_H ($D52^H$ and $N54^H$; blue) are stabilized by crystal contacts involving $R18^L$ of a crystallographically adjacent Fv complex (grey). Donor-acceptor distances (Å) for H-bonds and salt bridges are labelled. **(C)** Sidechain NH_2 region of ^{1}H-^{15}N HSQC spectra of 6D8 scFv in the presence of 3D7-MSP2$_{14\text{-}30}$ (blue) and FC27-MSP2$_{14\text{-}30}$ (red) showing the absence of chemical shift perturbations that are expected if E27 of 3D7 MSP2 interacts with the sidechain of $N54^H$ in solution.

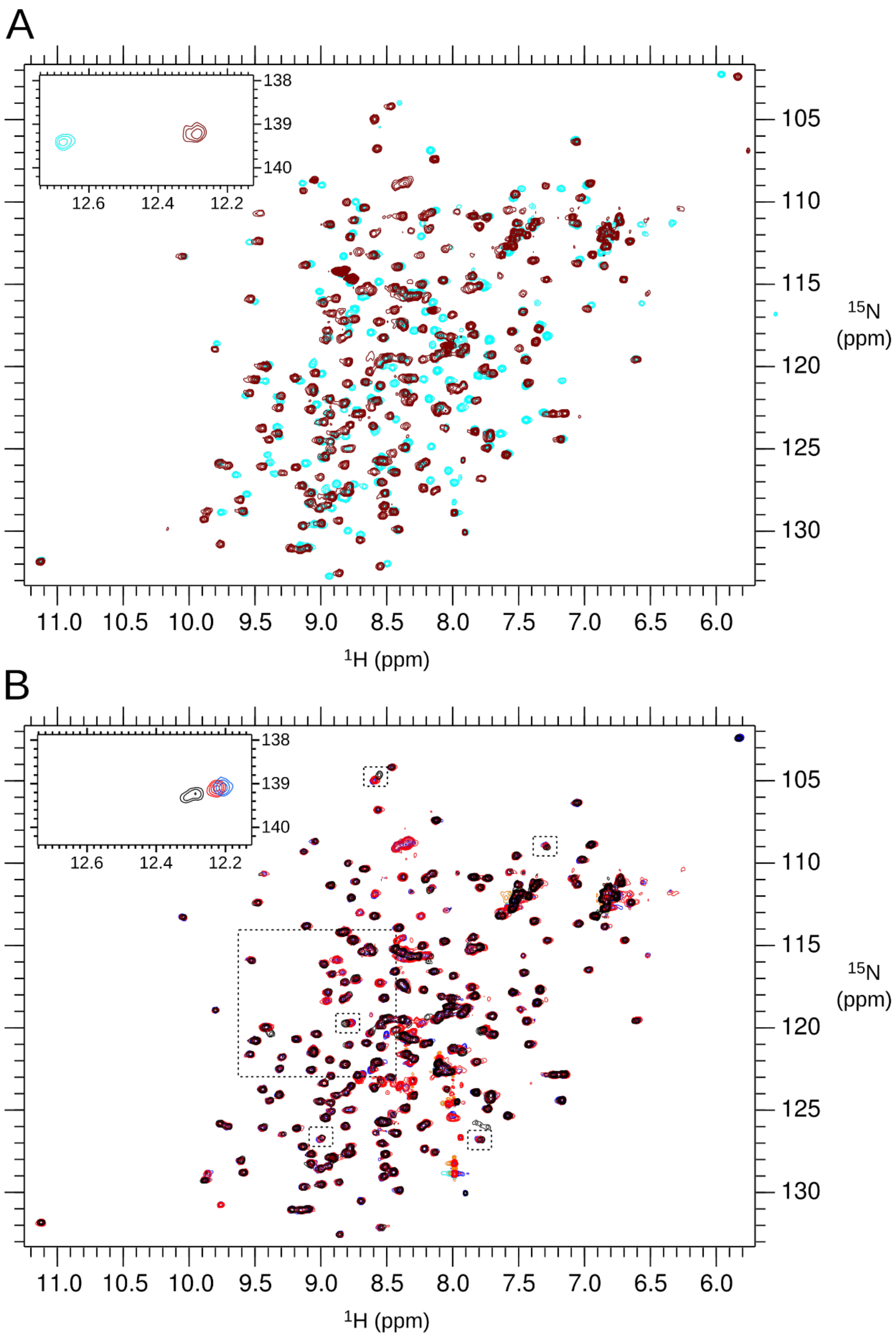

Figure S7. (A) Overlay of ^{1}H-^{15}N HSQC spectra of 6D8 scFv in the absence (cyan) and presence (brown) of MSP2$_{14-22}$ **(B)** Overlay of ^{1}H-^{15}N HSQC spectra of 6D8 scFv in the presence of MSP2$_{14-22}$ (black), FC27-MSP2$_{14-30}$ (blue) and 3D7-MSP2$_{14-30}$ (red). Spectral regions shown in Fig 4 (main text) are boxed, and an outlying Trp indole NH peak is inset.

```
                                          CDR1                 CDR2
IGHV1-78       QVQLQQSGDELVKPGASVKISCKVSGYTFTDHTIHWMKQRPEQGLEWIGYIYPRDGSTKY 60
6D8VH          QVQLQQSGDELVKPGASVKLSCTVSGFNIKDDFIHWMKQRPEQGLEWIGRIDPANGYTKY 60
               *******************:**.***:.:.*. **************** * * :* ***

               CDR2                                CDR3
IGHV1-78       NEKFKGKATLTADKSSSTAYMQLNSLTSEDSAVYFCAR---------------- 98
6D8VH          APKFQDKATMTADTSSNTAYLQLSSLASEDAAVYYCATYGVAYWGQGTLVTVSA 114
                 **:.***:***.**.***:**.**:***:***:**


                                        CDR1                 CDR2
IGKV3-4        DIVLTQSPASLAVSLGQRATISCKASQSVDYDGDSYMNWYQQKPGQPPKLLIYAASNLES 60
6D8VL          DIVLTQSPASLAVSLGQRATISCKASQSVDHDGDSYMNWFQQKPGQSPKLLIYAASNLES 60
               ******************************:********:******.*************

                                                  CDR3
IGKV3-4        GIPARFSGSGSGTDFTLNIHPVEEEDAATYYCQQSNEDPPTVLQG------ 105
6D8VL          GIPARFSGSGSGTDFTLNIHPVEEEDAATYYCQQTNEDPYTFGGGTKLEIK 111
               **********************************:**** *.  *
```

Figure S8. Alignment of the 6D8 V_H and V_L sequences (KM393285 and KM393286) obtained from the corresponding mouse hybridoma by PCR-amplification with the respective germline sequences IGHV1-78 and IGKV3-4 for V_H and V_L. The first V_H residue was replaced by the corresponding conserved Glu germline residue of IGHV1-78 to generate the 6D8 V_H construct used in this work. No changes were necessary for the V_L construct. The CDRs of each chain are highlighted.

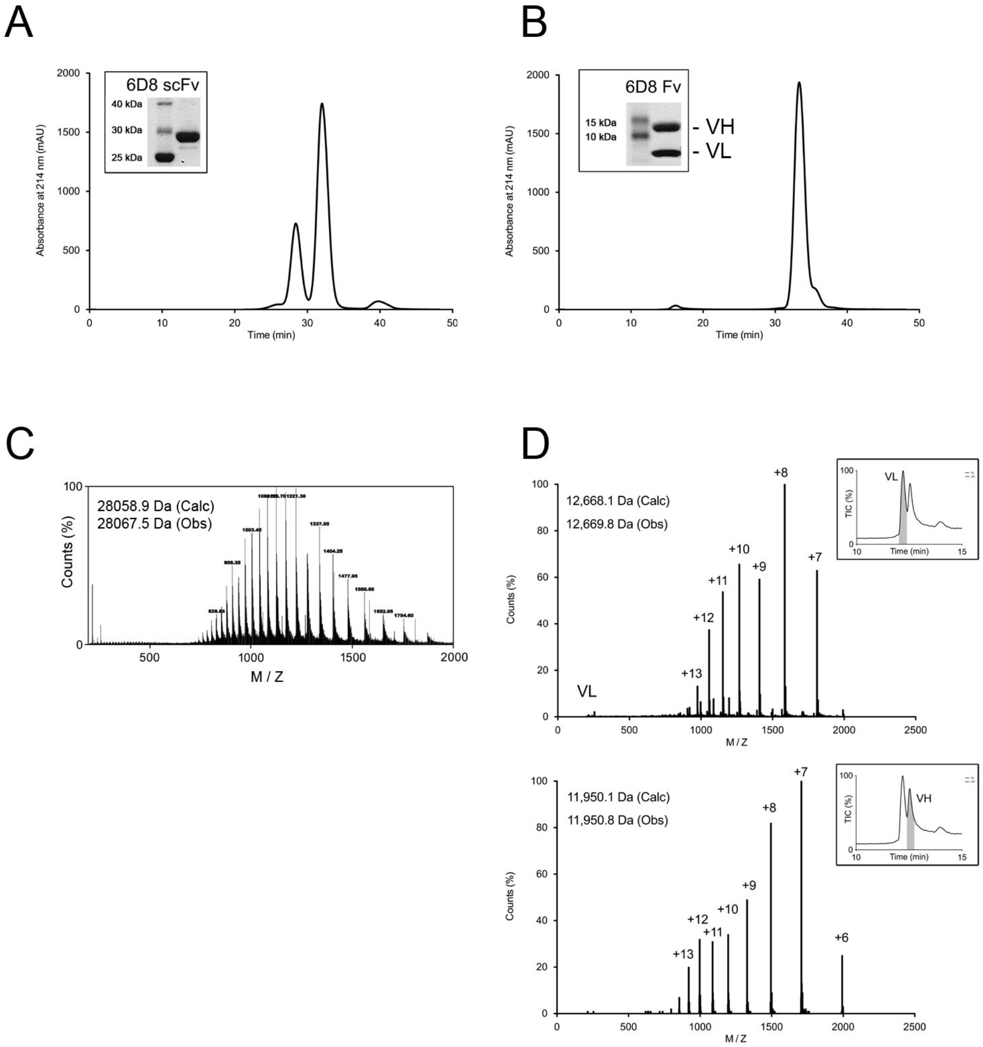

Figure S9. Purification profile of recombinant antibody fragments 6D8 scFv (A) and 6D8 Fv (B) used in this work. Antibody fragments were affinity purified, concentrated and freed from aggregates by gel filtration prior to structural and binding studies. The molecular masses observed for scFv (C) and Fv (D) were consistent with their calculated average masses detected by LCMS. The SDS-PAGE profile of each construct is presented in A and B.

References

1. Zhang, X. et al. Solution conformation, backbone dynamics and lipid interactions of the intrinsically unstructured malaria surface protein MSP2. *J Mol Biol* **379**, 105-121 (2008).
2. MacRaild, C.A., *et al*. Conformational dynamics and antigenicity in the disordered malaria antigen merozoite surface protein 2. *PLoS ONE* **(in press)**(2015).
3. Adda, C.G. et al. Antigenic characterization of an intrinsically unstructured protein, *Plasmodium falciparum* merozoite surface protein 2. *Infect Immun* **80**, 4177-85 (2012).
4. Adda, C.G. et al. *Plasmodium falciparum* merozoite surface protein 2 is unstructured and forms amyloid-like fibrils. *Mol Biochem Parasitol* **166**, 159-171 (2009).
5. Orlandi, R. & Güssow, D.H. Cloning immunoglobulin variable domains for expression by the polymerase chain reaction. *Proc Natl Acad Sci U S A* **24**, 527-31 (1989).
6. Rouet, R. et al. Expression of high-affinity human antibody fragments in bacteria. *Nat Protoc* **7**, 364-73 (2012).
7. Marley, J., Lu, M. & Bracken, C. A method for efficient isotopic labeling of recombinant proteins. *J Biomol NMR* **20**, 71-5 (2001).
8. Fields, G.B. & Noble, R.L. Solid phase peptide synthesis utilizing 9-fluorenylmethoxycarbonyl amino acids. *Int J Pept Protein Res* **35**, 161-214 (1990).
9. Schanda, P. & Brutscher, B. Very fast two-dimensional NMR spectroscopy for real-time investigation of dynamic events in proteins on the time scale of seconds. *J Am Chem Soc* **127**, 8014-5 (2005).
10. Kabsch, W. XDS. *Acta Crystallogr* **D66**, 125-132 (2010).
11. CCP4. The CCP4 suite: programs for protein crystallography. *Acta Crystallogr* **D50**, 760-763 (1994).
12. Brunger, A.T. Assessment of phase accuracy by cross validation: the free R value. Methods and applications. *Acta Crystallogr D Biol Crystallogr* **49**, 24-36 (1993).
13. McCoy, A.J., Grosse-Kunstleve, R.W., Storoni, L.C. & Read, R.J. Likelihood-enhanced fast translation functions. *Acta Crystallogr D Biol Crystallogr* **61**, 458-64 (2005).
14. Cohen, S.X. et al. ARP/wARP and molecular replacement: the next generation. *Acta Crystallogr D Biol Crystallogr* **64**, 49-60 (2008).
15. Adams, P.D. et al. PHENIX: a comprehensive Python-based system for macromolecular structure solution. *Acta Crystallogr* **D66**, 213-221 (2010).
16. Afonine PV et al. Towards automated crystallographic structure refinement with phenix.refine. . *Acta Cryst. D* **D68**, 352-367 (2012).
17. Emsley, P. & Cowtan, K. Coot: model-building tools for molecular graphics. *Acta Crystallogr D Biol Crystallogr* **60**, 2126-32 (2004).
18. Krissinel, E. & Henrick, K. Inference of macromolecular assemblies from crystalline state. *J Mol Biol* **372**, 774-97 (2007).
